# Hypertension among people living with human immunodeficiency virus receiving care at referral hospitals of Northwest Ethiopia: A cross-sectional study

**DOI:** 10.1371/journal.pone.0238114

**Published:** 2020-08-21

**Authors:** Alemu Gebrie

**Affiliations:** Department of Biomedical Science, School of Medicine, Debre Markos University, Debre Markos, Ethiopia; Addis Ababa University School of Public Health, ETHIOPIA

## Abstract

**Introduction:**

Hypertension among HIV positive patients in low- and middle-income countries has got little attention and data on the problem is limited in Ethiopia. Hence, this study aims to determine the magnitude of hypertension and its associated factors among HIV-positive patients receiving care at referral hospitals of Northwest Ethiopia

**Materials and methods:**

A cross-sectional study design was conducted to determine the burden of hypertension in patients living with HIV receiving care at referral hospitals of Northwest Ethiopia between November 2018 and May 2019. Four hundred seven randomly selected adult patients were included for the study. Using standardized questionnaire, sociodemographic, behavioral and clinical data were collected. Anthropometric parameters, fasting blood sugar as well as lipid profiles were determined. Bivariate and multivariate binary logistic regression analysis was performed.

**Result:**

A total of 407 study subjects with 98% response rate have been included in this study. The prevalence of hypertension was 14.0% (95% CI: 10.63,17.37). Elementary educational status as compared to no education [AOR (95% CI) 2.75 (1.12,6.75), p< 0.05], moderate monthly income compared to low [AOR (95% CI) 4.27 (2.09,8.73), p<0.01], waist circumference [AOR (95% CI) 4.27 (2.09,8.73), p<0.01], taking concomitant other drug therapy [AOR (95% CI) 5.72 (2.25,14.54), p<0.01] and duration of antiretroviral therapy [AOR (95% CI) 1.12 (1.04,1.20) were significantly associated with hypertension.

**Conclusion:**

Hypertension is not uncommon in patients living with HIV. Educational status, monthly income, waist circumference, concomitant drug therapy and duration of antiretroviral therapy are linked with hypertension. The finding pinpoints that health care providers should work up on risk factors to reduce the burden of hypertension among the patients.

## Background

Globally, it is estimated that there are about 37.9 million people living with HIV (PLHIV) [1]. Hypertension (HTN), characterized by persistently raised blood pressure, is the primary modifiable risk factor for cardiovascular diseases (CVDs) which are the leading causes of morbidity and mortality in PLHIV [2, 3]. HTN is a particular target among different non-AIDS chronic diseases [4]. Its trend has been increasing among PLHIV associated with substantially increased survivorship of the patients due to highly active antiretroviral therapy (HAART) [5, 6]. Some studies have also revealed the magnitude of hypertension in PLHIV to be higher than the general population [7, 8]. This increased risk is partly attributed to HIV related factors like long-term effects of HAART, hypercoagulation, premature atherosclerosis, activation of the immune system and elevated systemic inflammatory markers [8–13].

Researchers in public health as well as clinical streams have long been recognizing the importance of HTN among PLHIV. Plethora of studies have reported data on HTN among PLHIV in different countries and regions across the world [5, 14–26]. The reports vary from 4.0% to 67.0% by study population, area and time [25, 26]. Although the reports give useful data for clinical practitioners and public health experts to prevent and treat hypertension regionally, hypertension among HIV positive patients in low- and middle-income countries has gained a recent attention [27]. Data on the burden of hypertension among PLHIV have still been limited in Africa as well as Ethiopia, and the study area in particular.

Many risk factors such as older age, male gender, family history, ethnicity, longer duration of HIV infection, high viral load, smoking, alcohol use, comorbidity state, other drugs and substance use, low CD4 count, and obesity may be attributed to the high burden of HTN in HIV-infected subjects [21, 28]. In addition, certain drugs in ART regimen combinations have also been indicated to lead to hypertension among PLHIV [29, 30]. In support of this, a systematic review and meta-analysis of 39 studies enrolling about 44,903 HIV-infected patients revealed the risk of hypertension to be higher in PLHIV exposed to ART as compared to ART-naive patients [31]. With the current World Health Organization (WHO) “test and treat” recommendation, there are growing number of ART-exposed patients, and an increased burden of hypertension in PLHIV is inevitable [32]. On the other hand, a systematic review and meta-analysis from sub-Saharan Africa countries revealed lower blood pressure levels among PLHIV than uninfected adults [33]. A population-based study in South Africa also indicated that hypertension was less common among HIV-infected adults [34].

Generally, assessing the burden of hypertension among PLHIV are needed for decision-making as well as evidence-based planning to restructure and integrate tailored care of hypertension into HIV/AIDS management package. It is difficult to do this integration without having strong evidence on the burden of hypertension among HIV-infected patients and its risk factors. In view of the importance of the problem and the paucity of evidence on it, the main aim of this study was, therefore, to investigate the magnitude of hypertension and its associated risk factors among HIV-positive patients receiving care at referral hospitals of Northwest Ethiopia.

## Materials and methods

### Study design, setting, population and period

Hospital based cross-sectional study was carried out among adult PLHIV on HAART. The study was conducted between November 2018 and May 2019 among the patients attending the ART clinics of Debre Markos Referral Hospital (DMRH) and Felege Hiwot Referral Hospital (FHRH), two of the three referral hospitals in Northwest Ethiopia. The referral hospitals have been serving more than 10,000 PLHIV. Services in these ART clinics include: HIV testing and counseling, CD4 count tests and viral load so as to monitor the progress of the therapy, antiretroviral drugs dispensing, education and counselling to improve treatment adherence and to decrease defaulters as well as opportunistic infections follow-up. Whereas patients aged 18 years or older and signed an informed consent form to take part in the study were included in the study, pregnant women, and patients who were seriously ill and unable to respond were excluded from the study. In addition, patients on corticosteroids, oral contraceptives or other medications which may influence blood pressure were excluded.

### Sample size determination and sampling technique

By using the following single population proportion formula, the sample size (n) of this study was calculated. Considering the following parameters, 95% level of confidence level (z_α/2_ = 1.96), 5% of marginal error (d = 0.05), 6% non-response rate, and taking the prevalence (53%) of hypertension (p = 0.53, q = 1-p) in Ethiopian HIV-infected adults [23], the final sample size was determined to be 415. In addition, simple random sampling technique was used to select the study participants during their follow-up visits; the study participants were withdrawn from the total patients on follow up by lottery method from their registry.

### Variables of the study

Hypertension among HIV was the dependent variable of this study whereas diseases related factors like viral load, co-morbidities, duration of ART, WHO stage of the disease, duration HIV, presence of opportunistic infections; treatment related factors like concomitant drug therapy, type of ART regimens, duration of ART; Patient related factors such as age, gender, educational level, occupation, income status as well as patient’s behavioral related factors such as history of drug use, lifestyle of the patients were considered as predictor variables of the study.

The primary outcome of interest of the study was hypertension, which was defined according to WHO to be persistently elevated blood pressure, SBP≥140 mmHg and/or DBP ≥90 mmHg, or reported uses of antihypertensive medication [35]. Patients were grouped as smokers (yes/no), yes, if they smoke at least one cigarette for the last 12 months, and alcohol consumer (yes/no), yes, if they consume at least twice weekly of any alcoholic drinks. Regular physical activity was considered in patients reporting at least 30 min of intense physical activity, once a week or more to be categorized as yes [21, 22]. BMI was used to classify underweight: BMI<18.5 kg/m^2^, normal BMI: 18.5–24.99 kg/m^2^, and overweight: BMI: ≥25 kg/m^2^.

### Data collection

We used a questionnaire as a data collection tool adapted from WHO STEP wise approach to chronic disease risk factor surveillance [36, 37]. This tool consists of three parts for collecting data; the first is core and expanded sociodemographic and behavioral characteristics, the second involves core and expanded physical measurement, and the third consisted of biochemical measurements. After having informed consent from the study participants, sociodemographic data such as age, sex, educational status, marital status, monthly income, occupation and residence were collected. In addition, behavioral data like alcohol consumption habit, smoking and regular physical activity were collected both from patient interview and medical records of the patients using the questionnaire. Using the checklist, clinical data including viral load, comorbidity state, WHO stage, opportunistic infection, another drug therapy, type of ART regimen, other substance use and duration of HIV/AIDS were also collected from the patient’s medical record. Furthermore, anthropometric parameters (waist circumference, hip circumference, weight and height) and blood pressure (BP) data have been collected from the study participants. Finally, fasting blood sugar and lipid profiles have been determined. All the data were collected by trained and experienced clinical nurses and laboratory technologists with strict supervision of the principal investigator.

About 5 milliliters of fasting venous blood sample was taken from each study subject. After separation of serum from the whole blood, high-density lipoprotein cholesterol (HDL-c), low density lipoprotein cholesterol (LDL-c), total cholesterol (TC), triglyceride (TG), and fasting blood sugar (FBS) was analyzed at laboratory department of the referral hospitals, following the standard operating procedures. The blood specimen collection, separation of serum, and the laboratory analysis of the metabolites were done by experienced medical laboratory technologists working in the hospitals.

BP was measured by nurse professionals using a sphygmomanometer. The BP measurement was taken from the left arm, three times at 5 min interval after the patient had been sitting for at least 30 min. The average of the last two of three measurements was estimated and used in the analysis.

By using a digital balance with an attached height measurement, the body weight and height readings were taken to the nearest 0.1 kg and 0.1 cm, respectively. The height readings of the study participants were taken while they are facing directly ahead and the weight readings were obtained by placing the weighing balance instrument on a flat hard surface.

Hip and waist circumferences were also measured to a precision of 0.1 cm by using a tape that is non-stretchable. Hip circumference readings were taken at a point where maximum circumference can be obtained over the buttocks with a tape in a horizontal plane, touching the skin but not compressing it. Waist circumference was read with a flexible inelastic tape placed on the midpoint between the lower rib margin and the iliac crest in a perpendicular plane to the long axis of the body while subjects were standing and breathing normally. The European cutoff was used to determine the waist circumference measurements as per WHO. As per the guidelines, the elevated waist circumferences of female and male, respectively, are ≥80 and ≥ 94cm [38].

### Laboratory analysis

Blood samples were analyzed for FBS level and lipid profileS (TC, HDL-c, LDL-c and TG)) using ABX Pentra 400 machine. Enzymatic colorimetric assay method was used for the measurement of total cholesterol (CHODPAP method) and triglyceride (GPO-PAP method), while direct homogeneous enzymatic colorimetric assay technique was utilized for the measurement of HDL-c and LDLc. FBG level was measured by glucose oxidase method (GOD-PAP).

### Data analysis and interpretation

All data were cleaned, coded and entered into Epi-Data version 3.1 and exported to the Statistical package for Social Science (SPSS) version 24.0 for statistical analysis. Figures and tables were used for data presentation. Frequency distribution was used for presenting categorical data whereas mean ± standard deviation (SD) was used for continuous data. In addition, associations for the predictors and outcome variables were assessed using logistic regression analysis. All variables with P-value <0.25 in bivariate logistic regression analysis were entered into a multivariable logistic regression model to control confounding effects. Crude odds ratio (COR) and adjusted odds ratio (AOR) with 95% CI were reported. All statistical tests were two-sided, and a P-value of <0.05 was considered for statistical significance.

### Data quality assurance

The questionnaire was pretested by study populations at Finote Selam Hospital before data collection and subsequent modification has been made when any difficulty or doubt occurred. The collected data has been checked for the clarity, consistency and completeness. The data collection questionnaire was adapted from a standard tool and all variables were filled on the data extraction format daily. All the laboratory processes were handled by experienced health care professionals.

### Ethics approval and consent to participate

Ethics was approved from Debre Markos University, School of Medicine, Ethics committee with a letter serial number, SOM/338/33/19, and date, 1/18/2019. Then, permission was taken from the managers of hospitals by paraphrasing their approval signature on the letter before data collection starts. In addition, the study participants signed informed consent before data collection begins. Participants were informed about the objective of the work, potential minimal risks and benefits of the research. The investigators also kept the confidentiality of the information provided by the respondents by not using any patient identifier such as name.

## Result

### Socio‑demographic and behavioral characteristics of participants

Table 1 summarizes the sociodemographic characteristics of the study participants. A total of 407 study subjects with 98% response rate have been included in this study. The majority of the participants were female 246 (60.4%). The average age of the respondents was 38.6 ± 10.3 years with age range between 18 and 75 years. In addition, the majority of the respondents (71.3%) have attained at least primary level of education, and about half of the subjects (53.8%) were not married. About 17.4% of the participants were governmental employee in occupation, and the majority of the participants live in urban areas with most of them having monthly income of less than 2000 Ethiopian Birr. Finally, about 11.8% of the participants had history of alcohol drinking behavior whereas only 2.5% of them did smoke cigarette and majority of them (87%) did not do regular exercise.

**Table 1:** Socio-demographic and behavioral characteristic of people living with HIV receiving care at referral hospitals of Northwest Ethiopia, 2019 (n = 407).

Variables	Category	Frequency (%)
**Age (years)**	Mean± SD	38.64 ±10.29
**Sex**	Male	161 (39.6)
	Female	246 (60.4)
**Educational status**	No education	117 (28.7)
	Elementary	148 (36.4)
	High school	71 (17.4)
	Diploma	42 (10.3)
	Degree/above	29 (7.1)
**Marital status**	Single	44 (10.8)
	Married	188 (46.2)
	Separated	34 (8.4)
	Divorced	78 (19.2)
	Widowed	63 (15.5)
**Occupation**	Gov employee	71 (17.4)
	NG employee	22 (5.4)
	Self-employed	186 (45.7)
	Non-paid	27 (6.6)
	Student	17 (4.2)
	Farmer	34 (8.4)
	Homemaker	50 (12.3)
**Monthly income**	<2000 ETB	222 (54.5)
	2000–5000 ETB	150 (36.9)
	>5000 ETB	35 (8.6)
**Residence**	Urban	277 (68.1)
	Rural	130 (31.9)
**Physical exercise**	Yes	53 (13)
	No	354 (87)
**Smoking status**	Yes	10 (2.5)
	No	397 (97.5)
**Alcohol consumption**	Yes	48 (11.8)
	No	359 (88.2)

ETB: Ethiopian Birr; 1 US Dollar = 28.37.40 Ethiopian Birr on 1/31/2019; SD: Standard Deviation; Gov: Governmental; NG: Non-Governmental.

### Clinical as well as anthropometric related characteristics

In this study, it has been revealed that the prevalence of hypertension among people living with HIV was 14.0% (95% CI: 10.63,17.37) (Table 2). About one fifth of the participants were underweight and 16.5% of the patients were overweight /obese. Regarding to the regional distribution of fat, two fifth of the patients had waist circumference above the cut-off value and about 75% of the patients had high waist to hip ratio which is a better indicator of body fat distribution. The majority of the participants had viral load (copies/mL) less than 1000, and 19.7% of the subjects had opportunistic infection or comorbidity. In addition, about 11.3% of the HIV patients used drugs and substances other than ART mostly steroid drugs and contraceptives. Finally, most of the patients were in WHO clinical stage-I; 71.1% of the patients have HIV duration of more than five years, and TDF/3TC/EFV was the most frequent cART regimen (Table 2).

**Table 2:** Clinical and anthropometric related characteristics of people living with HIV receiving care at referral hospitals of Northwest Ethiopia, 2019 (n = 407).

Variable	Category	Frequency (%)
**Body Mass Index(kg/m2)**	Underweight	83 (20.3)
	Normal	257 (63.1)
	Overweight	59 (14.5)
	Obese	8 (2.0)
**Waist circumference**	High	168 (41.3)
	Normal	239 (58.7)
**Waist to hip ratio**	High	305 (74.9)
	Normal	102 (25.1)
**Viral load (copies/mL)**	Less than 1000	369 (90.7)
	More than 1000	38 (9.3)
**Comorbidity state**	Yes	65 (16)
	No	342 (84)
**WHO clinical stage**	Stage I	403 (99.0)
	Stage II	4 (1.0)
**Opportunistic Infection**	Yes	15 (3.7)
	No	392 (96.3)
**Another drug therapy**	Yes	33 (8.1)
	No	374 (91.9)
**Type of ART regimen**	TDF/3TC/EFV	186 (45.7)
	AZT/3TC/NVP	96 (23.6)
	TDF/3TC/NVP	42 (10.3)
	TDF/3TC/ATV/r	33 (8.1)
	AZT/3TC/EFV	14 (3.4)
	AZT/3TC/ ATV/r	36 (8.8)
**Other substance use**	Yes	13 (3.2)
	No	394 (96.8)
**Duration of living with HIV**	< 5 Years	77 (18.9)
	5–10 Years	178 (43.7)
	>10 Years	152 (37.3
**Hypertension**	Yes	57 (14.0)
	No	350 (86.0)

### Factors associated with hypertension among HIV/AIDS infected patients

Age, educational status, monthly income, BMI, WC, concomitant drug therapy, duration of HIV/AIDS, duration of cART, TG, FBS and comorbidity state had P value < 0.25 in the bivariate analysis. Therefore, these variables have been included in multivariate logistic regression analysis. Educational status, monthly income, WC, concomitant drug therapy and duration of cART were significantly associated with hypertension in HIV infected patients in the multivariate analysis.

Educational status was significantly associated with hypertension among HIV infected patients. Patients who attended education to elementary level were about 2.75 times more likely to have hypertension as compared to those with no education, [AOR (95% CI) 2.75 (1.12,6.75), p< 0.05]. HIV patients with monthly income of 2000–5000 ETB were at higher risk to have hypertension as compared to patients with monthly income of less than 2000 ETB [AOR (95% CI) 4.27 (2.09,8.73), p<0.01]. In addition, WC was significantly associated with hypertension in the HIV infected patients. For a centimeter increase in WC, the patients were 7% more likely to have hypertension, [AOR (95% CI) 1.07 (1.03,1.11), p< 0.05]. Moreover, concomitant drug therapy and duration of cART have shown statistically significant association with hypertension in the patients. Patients with concomitant drug therapy were about 5.72 times more likely to have hypertension as compared to their counterparts, [AOR (95% CI) 5.72 (2.25,14.54), p<0.01]. In addition, duration of cART was significantly associated with hypertension in the patients. For each year increase in duration of cART, the patients were 12% more likely to have hypertension, [AOR (95% CI) 1.12 (1.04,1.20), p< 0.05] (Table 3).

**Table 3:** Bivariate and multivariate analyses of socio-demographic, anthropometric and clinical risk factors for hypertension of people living with HIV receiving care at referral hospitals of Northwest Ethiopia, 2019 (n = 407).

Variable	Category	Hypertension		Bivariate analysis		Multivariate analysis	
		No	Yes	COR (95% CI)	P-value	AOR (95% CI)	P-value
**Age**				1.03 (1.00, 1.05)	0.050	1.02 (0.98,1.05)	0.374
**Educational status**	No education	40	4	1	-	1	-
	Elementary	162	26	3.18 (1.39,7.27)	0.006	2.75 (1.12,6.75)	0.027*
	High school	29	5	2.77 (1.07,7.16)	0.035	2.44 (0.86,6.89)	0.094
	Diploma	67	11	2.27 (0.74,6.99)	0.153	0.80 (0.23,2.78)	0.731
	Degree/above	52	11	1.57 (0.39,6.34)	0.525	0.22 (0.03,1.54)	0.126
**Monthly Income**	<2000 ETB	204	18	1	-	1	-
	2000–5000 ETB	115	35	3.45 (1.87,6.37)	0.000	4.27 (2.09,8.73)	0.000*
	>5000 ETB	31	4	1.46 (0.46,4.61)	0.516	2.46 (0.69,8.87)	0.167
**BMI**				1.06 (0.99,1.14)	0.119	0.97 (0.87,1.07)	0.544
**WC**				1.08 (1.05,1.12)	0.000*	1.07 (1.03,1.11)	0.000*
**Concomitant drug therapy**	Yes	20	13	4.88 (2.27,10.49)	0.000*	5.72 (2.25,14.54)	0.000*
	No	330	44	1	1	1	-
**Duration of living with HIV**				1.09 (1.00,1.18)	0.063	0.94 (0.81,1.08)	0.368
**Duration of cART**				1.11 (1.03,1.20)	0.007*	1.12 (1.04,1.20)	0.002*
**TG**				1.003 (1.001, 1.006	0.014*	1.002 (0.99,1.00)	0.239
**FBS**				1.01 (0.99,1.02)	0.199	1.00 (0.98, 1.01)	0.919
**Comorbidity state**	Yes	47	18	2.98 (1.57,5.63)	0.001*	1.08 (0.38,3.10)	0.882
	No	303	39	1	-	1	-

## Discussion

This study has been carried out with the objective of determining the magnitude and identifying associated factors of hypertension in HIV-infected patients on combined HAART. The overall magnitude of hypertension among PLHIV in this study was 14.0%, which is lower than the estimated prevalence of the general population of Ethiopia (31.2%) [39]. However, in line with previous studies [7, 8], the current finding is slightly higher than the result (12.5%) of a community based study in the study area [40]. The finding is also comparable with results of studies conducted in different countries including, 12.7% in Eastern Ethiopia [27], 17.1% in Northwest Ethiopia [41], 15.9% in Southern Ethiopia [42], 12.3% in Nigeria [19], 11.9% in Spain [43] and 13.5% in the United States [44]. However, the magnitude in this study is lower than the finding from related studies; 29.7% in Northeast Ethiopia [21], 34.4% Southwest Ethiopia, 29.9% in Zimbabwe [45], 43.0% in the United States [14] and 25.2% in a meta-analysis of prevalence of hypertension among people living with HIV [5]. On the other hand the result of this study is higher than some studies conducted in different parts of the world including Ethiopia [20, 26, 46, 47]. The variation could be due to differences in the HIV infection stages and duration, type of ART used and its duration or because of variations in the lifestyle and age distribution of the studied participants. In addition, the difference may also be due to differences in sample size and study design, hypertension definition, and the socioeconomic, demographic, ethnic and metabolic-related factors.

The link between HIV/AIDS and CVD is explained by several proposed mechanisms [48, 49]. Endothelial dysfunction resulting in increased CVD risk is contributed by chronic inflammation, platelet activation, and hypercoagulability mediated by HIV infection per se [50, 51]. The mechanism by which HIV induces endothelial dysfunction is proposed to be activation of white blood cells resulting in the secretion of cytokines, and a direct influence of tat and gp120 proteins released by HIV on the endothelium [52]. The other mechanism involves oxidative damage of the endothelium because of excess nitric oxide that reacts with oxygen radicals to producing peroxynitrite [53]. It has been shown that the use of HAART in PLHIV reduces the markers of coagulation and endothelial function [48–50].

Educational status, monthly income, WC, concomitant drug therapy and duration of cART were significantly associated with hypertension in HIV infected patients. Patients attending education to the level of elementary school and those with monthly income of 2000–5000 ETB were more likely to develop hypertension as compared to those with no education and monthly income of less than 2000 ETB, respectively. A possible justification for this positive association could be due to economic improvement facilitates access to some attractive unhealthy behaviors such as fast food-based diet and physical inactivity [54].

Concomitant drug therapy for other diseases was significantly associated with hypertension in the study subjects. This is due to the reason that the particularly used drugs in the patients such as corticosteroids, antidepressants and estrogens can induce either a persistent or transient blood pressure increment [55]. In addition, in line with other studies [56–59], WC was significantly associated with hypertension in the HIV infected patients. For a centimeter increase in WC, the patients were 7% more likely to have hypertension. The cellular mechanisms responsible for the association between metabolic or cardiovascular complications and WC are not well understood. However, different factors have been proposed. These hypothesized factors are sympathetic nervous system activation, genetic predisposition, insulin resistance and release of detrimental inflammatory adipocytokines by intra-abdominal adipose tissue as well as release of cortisol and angiotensinogen [60, 61]. Furthermore, the multivariate analysis of the present study indicated an independent association between duration of ART exposure and hypertension. For each year increase in duration of cART, the patients were 12% more likely to have hypertension. This finding is in line with other studies [27, 31, 62]. This association of the duration of cART and hypertension could be mediated directly through changes in endothelial function or it could be due to longevity as well as the age-associated comorbidities like ART-related changes in body composition and weight gain [21, 28].

## Limitation of the study

This study has limitations mainly related to the nature of cross-sectional study design; causality cannot be established. Stronger study designs like prospective cohort and clinical trials are then called for so as to identify the predictors of hypertension in the study subjects. Moreover, environmental factors like dietary factors as well as genetic factors have not been studied, and inclusion of HIV negative controls would have been better as a comparison group to minimize confounders. The study being limited to only two health institution, representativeness may also be affected.

## Conclusion and recommendation

Hypertension is not uncommon in PLHIV. Educational status, monthly income, waist circumference, concomitant drug therapy and duration of antiretroviral therapy were significantly associated with hypertension. The finding pinpoints that health care providers should work up on risk factors to reduce the burden of hypertension among the patients in order to prevent the risk of cardiovascular complications, and improve the health of PLHIV on ART.

## Abbreviations

3TC: Lamivudine
AOR: Adjusted Odds Ratio
ATV/r: Atazanavir
AZT: Zidovudine
BP: Blood Pressure
CVDs: Cardiovascular Diseases
EFV: Efavirenz
HAART: Highly Active Anti-Retro Viral Therapy
HDL: High Density Lipoprotein
HIV/AIDS: Human immunodeficiency virus/Acquired immunodeficiency syndrome
HTN: Hypertension
IDF: International Diabetes Federation
LDL: Low Density Lipoprotein
NVP: Nevirapine
SPSS: Statistical package for social sciences
TC: Total Cholesterol
TDF: Tenofovir
TG: Triglyceride
WC: Waist Circumference
WHO: World Health Organization
